# Supplementary material for: Digitalization impacts the COVID-19 pandemic and the stringency of government measures
Source: Sci Rep. 2022 Dec 14;12:21628. doi: 10.1038/s41598-022-24726-0 (PMC9749635; doi:10.1038/s41598-022-24726-0)
Supplement: Supplementary file 1 — Supplementary Information. [file 41598_2022_24726_MOESM1_ESM.pdf]

Supplementary Information

Digitalization impacts the COVID-19 pandemic and the stringency of government measures

Helen Heinrichs[1], Florian Mueller[1,2], Lucia Rohfleisch[1], Volkmar Schulz[1,2,3], Steven R. Talbot[4,†,*] & Fabian Kiessling[1,3,5,†,*]

1 Institute for Experimental Molecular Imaging, Center for Biohybrid Medical Systems, RWTH Aachen University Clinic, Aachen, Germany

2 Department of Physics of Molecular Imaging Systems, Institute for Experimental Molecular Imaging, Center for Biohybrid Medical Systems, RWTH Aachen University Clinic, Aachen, Germany

3 Fraunhofer Institute for Digital Medicine MEVIS, Bremen, Germany

4 Institute for Laboratory Animal Science, Hannover Medical School, Hannover, Germany

5 Comprehensive Diagnostic Center Aachen, RWTH Aachen University Clinic, Aachen, Germany

† these authors contributed equally

* Correspondences: fkiessling@ukaachen.de & talbot.steven@mh-hannover.de

Contents

Supplementary Methods 3

Supplementary Figure 1. Country-specific examples for determining the decline in new deaths pm in the post-peak period. 3

Supplementary Table 1. Regression model of interaction effects of DAI and its subgroups. 4

Supplementary Figure 2. Classification of thresholds in DAI. 4

Supplementary Note: Search strategy 5

Supplementary Table 2. Eligibility criteria. 6

Supplementary Table 3. Preferred Reporting Items for Systematic Reviews and Meta-Analyses extension for Scoping Reviews (PRISMA-ScR) Checklist. 7

Supplementary Results 9

Supplementary Figure 3. Flowchart of screening and selection process. 9

Supplementary Table 4. List of included papers (n=70). 10

Supplementary Figure 4. Distribution of first authors' residence. 15

Supplementary Figure 5. Distribution of countries evaluated in the included articles. 15

Supplementary Table 5. Data synthesis. 16

Supplementary Figure 6. Occurrence of keywords and themes within the scoping review. 18

Supplementary Figure 7. Distribution of digital adoption by income group. 19

Supplementary Table 6. Post-hoc comparison of means between income groups. 19

Supplementary References 20

Supplementary Methods

Country-specific examples for determining the decline in new deaths pm

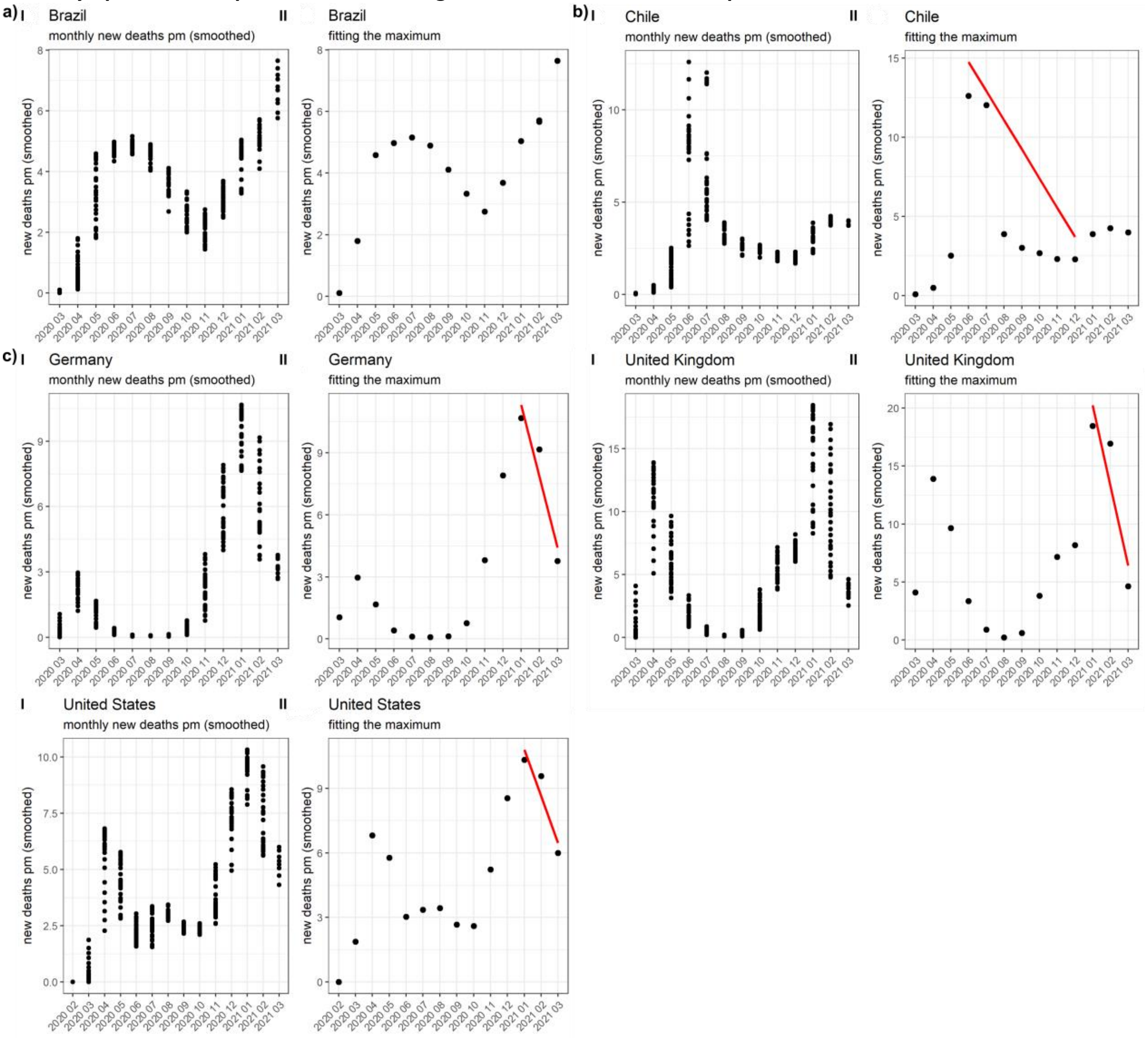

Supplementary Figure 1. Country-specific examples for determining the decline in new deaths pm in the post-peak period. The post-peak period describes the time and duration at which the spread of deaths has dropped below the peak. Linear regression was fit on the monthly data between the peak maximum and the maximum of the minimum that followed the peak. The period after the peak was determined separately for each country, and the slope values were finally included in the linear regression models. Countries were excluded if they a) were unable to reduce their death rates, b) experienced an increase in death rates that exceeded half of the peak height, or c) reached the peak at the end of the observation time (March 2021). Post-peak periods were analyzed for each country separately and, therefore, differed in length.

Determining thresholds and subgroups in DAI

Supplementary Table 1 presents the results of the treatment contrasts of the interaction of DAI and its three subgroups (low, medium, and high). This step is undertaken as an additional step after noticing three trends in data when plotting the SI_{mean} against DAI. The DAI:subgroup interaction shows a significant effect (β=-109.44, *p*<0.05). Therefore, DAI slope is dependent on the subgroups. This also indicates that the effect of a country's digitalization depends on its relative position within the DAI scale.

Supplementary Table 1. Regression model of interaction effects of DAI and its subgroups.

	**β**	**Std. Error of β**	**t**	***p-value***
Constant	38.61	10.65	3.627	0.000**
**DAI**	54.18	36.95	1.466	0.145
**medium**	32.56	14.28	2.280	0.024*
**high**	63.74	27.23	2.341	0.021*
**DAI:medium**	-64.79	40.84	-1.586	0.115
**DAI:high**	-109.44	49.22	-2.224	0.028*

* *p*≤0.05; ** *p*≤0.001;

Notes: The regression coefficient (β) is the degree of change in the outcome variable for every one-unit change in the predictor variable. The t-statistic is the regression coefficient divided by its standard error. Digital adoption is expressed in arbitrary units of DAI. The *Constant* in this regression analysis represents the mean value of the low DAI subgroup (the intercept).

Supplementary Fig. 2 shows the subgroup thresholds [0.36; 0.68], obtained from the n=61 inferential interaction analyses with linear regression in the range DAI [0.2: 0.8]. The thresholds discriminate the changes in trend in the data and determine the three DAI subgroups (low, medium, and high). In the plot, each bubble represents the p-value from one of the interaction analyses. We considered the continuous flow of a *p*≤0.05 to code for the medium subgroup.

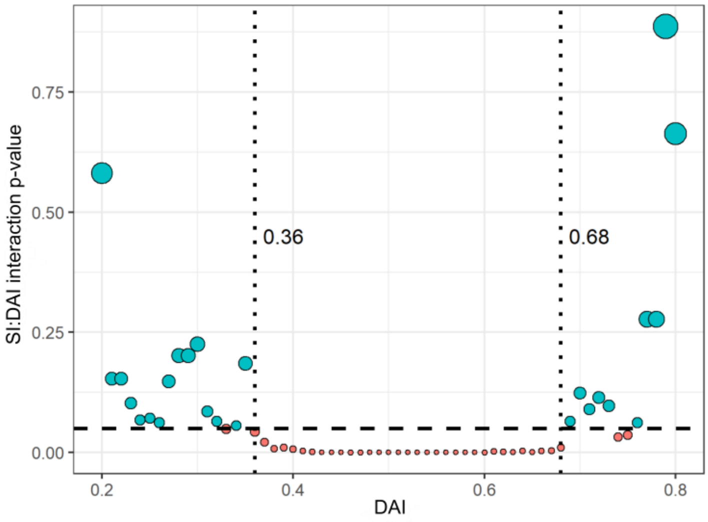

Supplementary Figure 2. Classification of thresholds in DAI. We observed three trends in DAI:SI interaction, where the p-value changed from *p*≤0.05 (red dots) to *p*≥0.05 (turquoise dots). The change in p-values determined the classification of the DAI into three categories (low, medium, and high). The dotted line shows the breaking points in data, respectively, thresholds of the three DAI categories [0.36; 0.68]. The different dot sizes represent the SI:DAI interaction p-values.

Supplementary Note: Search strategy

Search strategy used for PubMed (n=2,082).
("Coronavirus"[MeSH Terms] OR "Coronavirus"[Title/Abstract] OR "Coronaviruses"[Title/Abstract] OR "corona virus"[Title/Abstract] OR "sars-cov-2"[MeSH Terms] OR "sars-cov-2"[Title/Abstract] OR "Covid"[Title/Abstract] OR "Covid-19"[MeSH Terms] OR "Covid-19"[Title/Abstract] OR "Covid19"[Title/Abstract] OR "Coronaviridae"[MeSH Terms] OR "Coronaviridae"[Title/Abstract] OR "Coronavirinae"[Title/Abstract] OR "Betacoronavirus"[MeSH Terms] OR "Betacoronavirus"[Title/Abstract] OR "Betacoronaviruses"[Title/Abstract] OR "Ncov"[Title/Abstract] OR "pandemics"[MeSH Terms] OR "pandemic*"[Title/Abstract]) AND ("digit*"[Title/Abstract]) AND ("2020/01/01"[Date - Publication] : "3000"[Date - Publication])

Search strategy used for Web of Science (n=2,930).
((TS=(covid OR COVID-19 OR COVID19 OR coronavirus OR “corona virus” OR “coronavirus disease 2019” OR “SARS-CoV-2” OR nCoV OR pandemics OR coronaviridae OR coronavirinae OR betacoronavirus)) AND (TS=(digit*))) AND (DOP=(2020-01-01/2021-05-03))

Eligibility criteria

Supplementary Table 2. Eligibility criteria. Inclusion and exclusion characteristics of the studies included in the scoping review.

Type of publication and study design	- Studies published in a peer-reviewed scientific journal. We exclude articles that are in-press or pre-prints.<br>- Original research, reviews articles, and perspectives. We exclude news, rapid reports and communications, comments, conference abstracts, letters, policy briefs, and guidance, editorials, clinical studies, and articles where we failed to access full text.
Population	- All humans
Context	- The context of this paper will be global
Language restrictions	- All English-language papers
Publication date	- Articles published from 01/01/2020 to 03/05/2021
Interventions, exposures	- All COVID-19 related (such as clinical features of COVID-19, investigations relating to COVID-19, outcomes relating to COVID-19, associations with COVID-19 incidence, mortality)<br>- Descriptions of and/or applications of digital health technology solutions in the context of COVID-19 OR in the context of population-level public health interventions (it may refer to interventions that were at stake before the pandemic started)
Outcomes	- Due to the broad nature of this scoping review, outcome measures analyzing any topic related to COVID-19 and its intersection with digitalization will be included.<br><br>This will include but is not limited to:<br>- COVID-19 outcomes, disease frequencies, and epidemiology (mortality, morbidity, incidence, prevalence, deaths, …)<br>- Digital technologies used in the COVID-19 pandemic<br>- Digital technologies used before the COVID-19 pandemic<br>- Administrative outcomes (government responses, infrastructure/organization of hospitals, utilization of digital technologies)<br>- Equipment/safety-related (intensive care units, access to hospitals and operating units)<br>- Countries' digital performance (access to digital technologies; adoption, preparedness, and readiness for digital development; digital infrastructure)
Sort and prioritize your exclusion criteria per selection phase (optional)	Selection phase: title/abstract screening<br>1. Not COVID-19 AND/OR digit* related<br>2. Not English<br>3. Wrong population<br>4. Wrong publication type/study design<br><br>Selection phase: full-text screening<br>1. No full text is accessible<br>2. Not (primarily) COVID-19 AND/OR digit* related<br>3. Off-topic: Articles that did primarily focus on digital tools for mitigating COVID-19<br>4. Wrong outcomes<br>5. Wrong publication type/study design

Preferred Reporting Items for Systematic Reviews and Meta-Analyses extension for Scoping Reviews

Supplementary Table 3. Preferred Reporting Items for Systematic Reviews and Meta-Analyses extension for Scoping Reviews (PRISMA-ScR) Checklist.

SECTION	ITEM	PRISMA-ScR CHECKLIST ITEM	REPORTED ON PAGE #
TITLE			
Title	1	Identify the report as a scoping review.	We did not include this information in the title, as the scoping review was one of three methodologies applied.
ABSTRACT			
Structured summary	2	Provide a structured summary that includes (as applicable): background, objectives, eligibility criteria, sources of evidence, charting methods, results, and conclusions that relate to the review questions and objectives.	1
INTRODUCTION			
Rationale	3	Describe the rationale for the review in the context of what is already known. Explain why the review questions/objectives lend themselves to a scoping review approach.	2
Objectives	4	Provide an explicit statement of the questions and objectives being addressed with reference to their key elements (e.g., population or participants, concepts, and context) or other relevant key elements used to conceptualize the review questions and/or objectives.	12
METHODS			
Protocol and registration	5	Indicate whether a review protocol exists; state if and where it can be accessed (e.g., a Web address); and if available, provide registration information, including the registration number.	n.a.
Eligibility criteria	6	Specify characteristics of the sources of evidence used as eligibility criteria (e.g., years considered, language, and publication status), and provide a rationale.	Supplementary Table 2
Information sources*	7	Describe all information sources in the search (e.g., databases with dates of coverage and contact with authors to identify additional sources), as well as the date the most recent search was executed.	12
Search	8	Present the full electronic search strategy for at least 1 database, including any limits used, such that it could be repeated.	Supplementary Note
Selection of sources of evidence†	9	State the process for selecting sources of evidence (i.e., screening and eligibility) included in the scoping review.	Supplementary Table 2
Data charting process‡	10	Describe the methods of charting data from the included sources of evidence (e.g., calibrated forms or forms that have been tested by the team before their use, and whether data charting was done independently or in duplicate) and any processes for obtaining and confirming data from investigators.	12-13
Data items	11	List and define all variables for which data were sought and any assumptions and simplifications made.	Supplementary Table 4
Critical appraisal of individual sources of evidence§	12	If done, provide a rationale for conducting a critical appraisal of included sources of evidence; describe the methods used and how this information was used in any data synthesis (if appropriate).	n.a.
Synthesis of results	13	Describe the methods of handling and summarizing the data that were charted.	12-13
RESULTS			
Selection of sources of evidence	14	Give numbers of sources of evidence screened, assessed for eligibility, and included in the review, with reasons for exclusions at each stage, ideally using a flow diagram.	Supplementary Fig. 3

SECTION	ITEM	PRISMA-ScR CHECKLIST ITEM	REPORTED ON PAGE #
Characteristics of sources of evidence	15	For each source of evidence, present characteristics for which data were charted and provide the citations.	Supplementary Table 3, Supplementary Fig. 4 & 5
Critical appraisal within sources of evidence	16	If done, present data on critical appraisal of included sources of evidence (see item 12).	n.a.
Results of individual sources of evidence	17	For each included source of evidence, present the relevant data that were charted that relate to the review questions and objectives.	n.a.
Synthesis of results	18	Summarize and/or present the charting results related to the review questions and objectives.	Supplementary Table 3
DISCUSSION			
Summary of evidence	19	Summarize the main results (including an overview of concepts, themes, and types of evidence available), link to the review questions and objectives, and consider the relevance to key groups.	3-8 & Supplementary Results
Limitations	20	Discuss the limitations of the scoping review process.	10
Conclusions	21	Provide a general interpretation of the results with respect to the review questions and objectives, as well as potential implications and/or next steps.	9-11
FUNDING			
Funding	22	Describe sources of funding for the included sources of evidence, as well as sources of funding for the scoping review. Describe the role of the funders of the scoping review.	19

JBI = Joanna Briggs Institute; PRISMA-ScR = Preferred Reporting Items for Systematic reviews and Meta-Analyses extension for Scoping Reviews.

* Where *sources of evidence* (see second footnote) are compiled from, such as bibliographic databases, social media platforms, and Web sites.

† A more inclusive/heterogeneous term used to account for the different types of evidence or data sources (e.g., quantitative and/or qualitative research, expert opinion, and policy documents) that may be eligible in a scoping review as opposed to only studies. This is not to be confused with *information sources* (see first footnote).

‡ The frameworks by Arksey and O'Malley (6) and Levac and colleagues (7) and the JBI guidance (4, 5) refer to the process of data extraction in a scoping review as data charting.

§ The process of systematically examining research evidence to assess its validity, results, and relevance before using it to inform a decision. This term is used for items 12 and 19 instead of "risk of bias" (which is more applicable to systematic reviews of interventions) to include and acknowledge the various sources of evidence that may be used in a scoping review (e.g., quantitative and/or qualitative research, expert opinion, and policy document).

From: Tricco AC, Lillie E, Zarin W, O'Brien KK, Colquhoun H, Levac D, et al. PRISMA Extension for Scoping Reviews (PRISMAScR): Checklist and Explanation. Ann Intern Med. 2018;169:467–473. doi: 10.7326/M18-0850.

Supplementary Results

Flowchart of study selection process

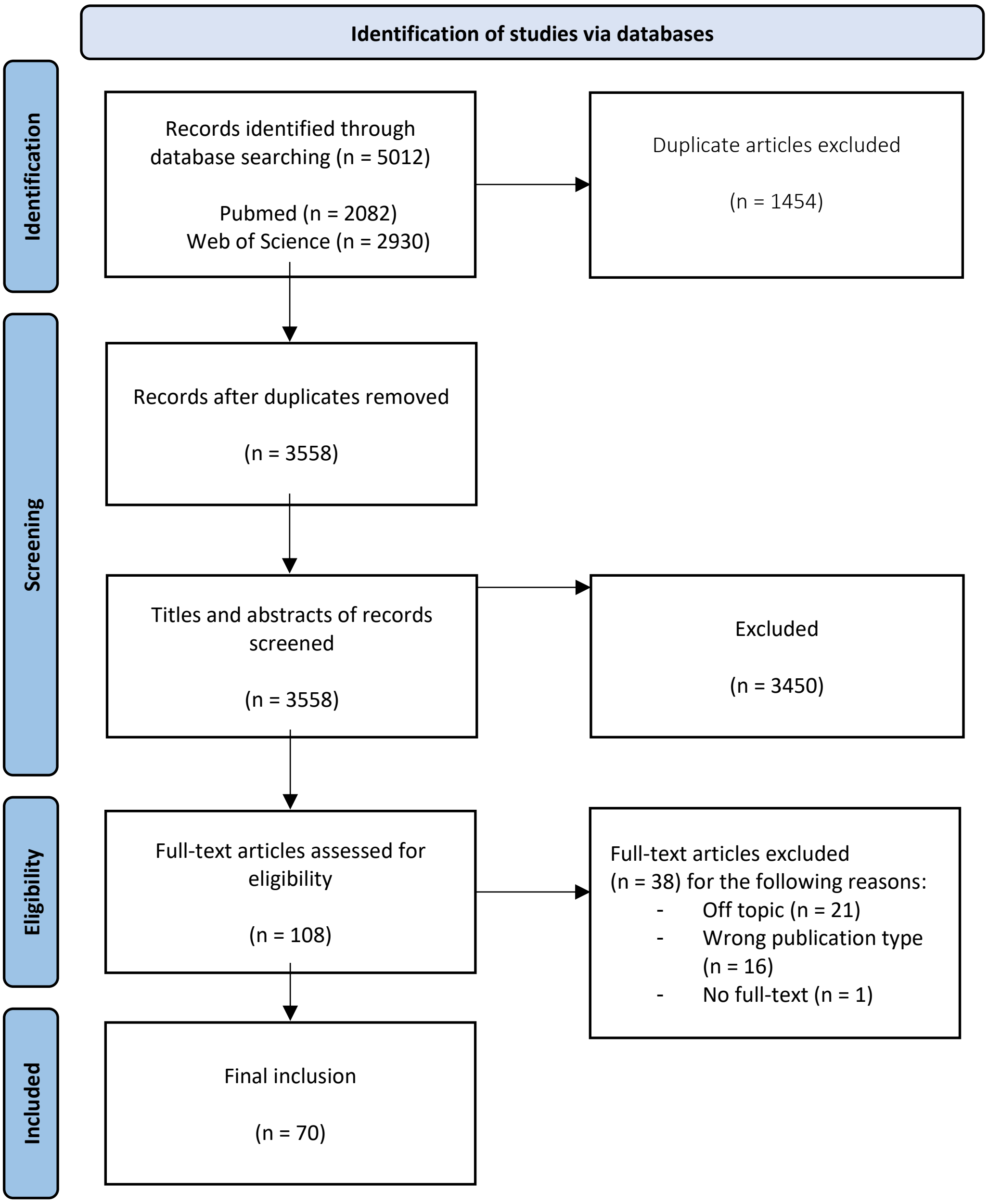

Supplementary Figure 3. Flowchart of screening and selection process.

Data extraction table

Supplementary Table 4. List of included papers (n=70).

First author	Publication Year	Title (type of article)	Description
Abdel-Basset, M. [1]	2020	An intelligent framework using disruptive technologies for COVID-19 analysis (original research)	The authors describe disruptive technologies and their benefits to combat COVID-19.
Abueg, M. & Hinch, R. [2]	2021	Modeling the effect of exposure notification and non-pharmaceutical interventions on COVID-19 transmission in Washington state (original research)	The influence of notifications combined with other non-pharmaceutical interventions to combat COVID-19 is analyzed.
Aggarwal, L. [3]	2020	Multi-criterion Intelligent Decision Support system for COVID-19 (original research)	Various technologies to combat COVID-19 in India are evaluated.
Alexiadis, A. [4]	2020	Simulation of pandemics in real cities: enhanced and accurate digital laboratories (original research)	The authors develop a framework to simulate the spread of infectious diseases in Birmingham and Bogotá.
Allam, M. & Cai, S. [5]	2020	COVID-19 Diagnostics, Tools, and Prevention (review article)	Recent trends in pandemic biology, diagnostics methods, prevention tools, and policies to combat COVID-19 are evaluated.
Almalki, A. [6]	2021	Health Apps for Combating COVID-19: Descriptive Review and Taxonomy (review article)	Mobile phone applications to combat COVID-19 are presented.
Anthony, B. [7]	2021	Integrating telemedicine to support digital health care for the management of COVID-19 pandemic (review article)	The authors discuss the benefits of telemedicine to combat COVID-19 worldwide.
Anthony, B. [8]	2020	Implications of telehealth and digital care solutions during COVID-19 pandemic: a qualitative literature review (review article)	The authors report on types of telehealth and digital care, policies, and the influence of telehealth and digital care solutions in countries worldwide.
Anttiroiki, A. V. [9]	2021	Successful Government Responses to the Pandemic: Contextualizing National and Urban Responses to the COVID-19 Outbreak in East and West (original research)	Various national strategies to combat COVID-19 are evaluated.
Asadzadeh, A. [10]	2021	A scope of mobile health solutions in COVID-19 pandemics (review article)	The authors analyze various mobile-health technologies to combat COVID-19.
Basellini, U. [11]	2021	Linking excess mortality to mobility data during the first wave of COVID-19 in England and Wales (original research)	The effects of mobility reductions in England and Wales between February and August 2020 are analyzed.
Behar, J. A. [12]	2020	Remote health diagnosis and monitoring in the time of COVID-19 (review article)	The authors evaluate remote health monitoring initiatives taken in 20 states.
Boeing, P. [13]	2021	Decoding China's COVID-19 'virus exceptionalism': Community-based digital contact tracing in Wuhan (original research)	The authors critically address China's exceptionalism in managing the COVID-19 pandemic with the example of the city Wuhan.
Budd, J. [14]	2020	Digital technologies in the public-health response to COVID-19 (review article)	Digital technologies to combat COVID-19 are analyzed in public health response to the pandemic.
Cencetti, G. & Santin, G. [15]	2021	Digital proximity tracing on empirical contact networks for pandemic control (original research)	The impact of digital contact tracing in combatting COVID-19 is evaluated using a framework based on contact data.

Chatterjee, P. [16]	2020	Internet of Things and Artificial Intelligence in Healthcare During COVID-19 Pandemic-A South American Perspective (review article)	The digital healthcare in South America during the COVID-19 pandemic is discussed.
Chaudhary, Y. [17]	2021	Digital Warfare Against COVID-19: Global Use of Contact-Tracing Apps (review article)	Contact tracing apps worldwide are evaluated regarding their safety, privacy, and efficacy.
Elavarasan, R. M. [18]	2021	A hover view over effectual approaches on pandemic management for sustainable cities - The endowment of prospective technologies with revitalization strategies (original research)	The authors analyze the characteristics of the pandemic as well as the role of technologies to manage it and lessons learnt.
Faraj, S. [19]	2021	Unto the breach: What the COVID-19 pandemic exposes about digitalization (original research)	Data are presented on how the COVID-19 pandemic challenges digitalization issues.
Fariniuk, T. M. D. [20]	2020	Smart cities and the pandemic: digital technologies on the urban management of Brazilian cities (original research)	The use of digital tools in Brazilian cities to combat COVID-19 is explored.
Ferretti, L. & Wymant, C. [21]	2020	Quantifying SARS-CoV-2 transmission suggests epidemic control with digital contact tracing (original research)	Various transmission routes of COVID-19 were investigated.
Golinelli, D. & Boetto, E. [22]	2020	Adoption of Digital Technologies in Health Care During the COVID-19 Pandemic: Systematic Review of Early Scientific Literature (review article)	Digital solutions to mitigate the impact of COVID-19 on individuals and the health systems are evaluated.
Gunasekeran, D. V. & Tseng, R. M. W. W. [23]	2021	Applications of digital health for public health responses to COVID-19: a systematic scoping review of artificial intelligence, telehealth and related technologies (review article)	Digital health solutions for public health responses are discussed.
He, Z., Zhang, C. J. P. & Huang, J. [24]	2020	A New Era of Epidemiology: Digital Epidemiology for Investigating the COVID-19 Outbreak in China (perspective article)	In this viewpoint, the authors estimate the disease spread patterns related to human activities.
Heo, K. [25]	2020	Searching for Digital Technologies in Containment and Mitigation Strategies: Experience from South Korea COVID-19 (review article)	Field applications of governmental interventions to combat COVID-19 are investigated.
Hernández-Orallo, E. [26]	2020	Evaluating the Effectiveness of COVID-19 Bluetooth-Based Smartphone Contact Tracing Applications (original research)	Newly developed contact tracing mobile applications to combat COVID-19 are evaluated.
Hernández-Orallo, E. [27]	2020	Evaluating How Smartphone Contact Tracing Technology Can Reduce the Spread of Infectious Diseases: The Case of COVID-19 (original research)	Contact tracing technologies and their impact on controlling infectious diseases are studied.
Huang, Z. [28]	2020	Performance of Digital Contact Tracing Tools for COVID-19 Response in Singapore: Cross-Sectional Study (original research)	The authors analyze the performance of the contact tracing app "Trace Together" and compares it to a wearable tag-based real-time locating system.
Islam, M. N. [29]	2020	A Systematic Review of the Digital Interventions for Fighting COVID-19: The Bangladesh Perspective (review article)	The authors analyze digital interventions to combat COVID-19 in Bangladesh and compare them to other countries.
Javaid, M. [30]	2020	Industry 4.0 technologies and their applications in fighting COVID-19 pandemic (review article)	Technologies of Industry 4.0 and their applications to combat COVID-19 are evaluated.

Javaid, M. [31]	2020	Industry 5.0: Potential Applications in COVID-19 (review article)	Industry 5.0 technologies that can be used to combat COVID-19 are explored.
Jian, S. W. [32]	2020	Contact tracing with digital assistance in Taiwan's COVID-19 outbreak response (perspective article)	The authors developed a centralized contact tracing system to combat COVID-19.
Jung, S. Y. [33]	2020	How IT preparedness helped to create a digital field hospital to care for COVID-19 patients in S. Korea (perspective article)	The impact of IT preparedness on creating a digital field hospital in South Korea is analyzed.
Kalhori, S. R. N. [34]	2021	Digital Health Solutions to Control the COVID-19 Pandemic in Countries With High Disease Prevalence: Literature Review (review article)	The application of digital technologies to combat COVID-19 in the ten countries with the highest prevalence of the disease is investigated.
Khan, Z. H. [35]	2020	Robotics Utilization for Healthcare Digitization in Global COVID-19 Management (original research)	The role of robotics in healthcare regarding the mastery of COVID-19 is studied.
Kogan, N. E., Clemente, L. & Liautaud, P. [36]	2021	An early warning approach to monitor COVID-19 activity with multiple digital traces in near real time (original research)	Digital data streams are analyzed regarding their role as indicators of COVID-19 activity.
Król, K. [37]	2021	The Quality of Infectious Disease Hospital Websites in Poland in Light of the COVID-19 Pandemic (original research)	The authors evaluate hospital websites in Poland in matters regarding COVID-19.
Kucharski, A. J. [38]	2020	Effectiveness of isolation, testing, contact tracing, and physical distancing on reducing transmission of SARS-CoV-2 in different settings: a mathematical modelling study	The effectiveness of COVID-19 transmission reduction strategies is estimated.
Lai, S. H. S. [39]	2020	The experience of contact tracing in Singapore in the control of COVID-19: highlighting the use of digital technology (review article)	Forms of contact tracing, especially new (digital) technology innovations used in Singapore to combat COVID-19, are evaluated.
Leung, K. & Wu, J. T. [40]	2021	Real-time tracking and prediction of COVID-19 infection using digital proxies of population mobility and mixing (original research)	The authors develop a framework that quantifies disease transmission models with age-specific digital mobility data.
Liaw, S. T. [41]	2021	Primary Care Informatics Response to Covid-19 Pandemic: Adaptation, Progress, and Lessons from Four Countries with High ICT Development (review article)	The authors evaluate the response to COVID-19 in Australia, Canada, the United Kingdom and the United States regarding primary care informatics.
Lin, L. [42]	2020	Combat COVID-19 with artificial intelligence and big data (perspective article)	The use of artificial intelligence and big data to combat COVID-19 in China (Mainland and Hongkong), Taiwan, and South Korea are evaluated.
Mahdi, A. [43]	2021	OxCOVID19 Database, a multimodal data repository for better understanding the global impact of COVID-19 (original research)	The Oxford COVID-19 Database is described.
Martin, T. [44]	2020	Demystifying COVID-19 Digital Contact Tracing: A Survey on Frameworks and Mobile Apps (review article)	The authors analyze the most concrete contact tracing architectures worldwide.
Meijer, A. [45]	2020	The COVID-19-crisis and the information polity: An overview of responses and discussions in twenty-one countries from six continents (review article)	The governmental responses to COVID-19 in 21 countries are compared, especially regarding their information policy.

Nachega, J. B. [46]	2021	Contact Tracing and the COVID-19 Response in Africa: Best Practices, Key Challenges, and Lessons Learned from Nigeria, Rwanda, South Africa, and Uganda (perspective article)	The COVID-19 contact tracing in Nigeria, Rwanda, South Africa and Uganda is evaluated.
Nageshwaran, G. [47]	2021	Review of the role of big data and digital technologies in controlling COVID-19 in Asia: Public health interest vs. privacy (review article)	The authors evaluate the digital tools used to combat COVID-19 in Taiwan, South Korea, China and Singapore.
Nuzzo, A. [48]	2020	Universal Shelter-in-Place Versus Advanced Automated Contact Tracing and Targeted Isolation: A Case for 21st-Century Technologies for SARS-CoV-2 and Future Pandemics (original research)	Digital contact tracing and shelter-in-place actions regarding their effect on the spread of COVID-19 are discussed.
O'Connor, H. [49]	2021	For the greater good? Data and disasters in a post-COVID world (research article)	The difficulties of data usage during the COVID-19 pandemic in Taiwan and New Zealand are addressed.
Reddick, C. G. [50]	2020	Determinants of broadband access and affordability: An analysis of a community survey on the digital divide (original research)	The digital divide is analyzed in a case study in San Antonio.
Rodríguez, P. [51]	2021	A population-based controlled experiment assessing the epidemiological impact of digital contact tracing (original research)	The authors conducted a population-based controlled experiment in La Gomera (Canary Islands) to analyze the impact of the Radar COVID mobile application.
Salathé, M. [52]	2020	Early evidence of effectiveness of digital contact tracing for SARS-CoV-2 in Switzerland (original research)	Digital contact tracing applications available in Switzerland are reported.
Schlosser, F. [53]	2020	COVID-19 lockdown induces disease-mitigating structural changes in mobility networks (original research)	The structural mobility changes during the COVID-19 pandemic in Germany are analyzed.
Scott, B. K. [54]	2020	Advanced Digital Health Technologies for COVID-19 and Future Emergencies (review article)	The authors evaluate digital health technologies useful to combat COVID-19.
Shamil, M. S. [55]	2020	An Agent-Based Modeling of COVID-19: Validation, Analysis, and Recommendations (research article)	The authors use an agent-based model to simulate the spread of COVID-19.
Skoll, D. [56]	2020	COVID-19 testing and infection surveillance: Is a combined digital contact-tracing and mass-testing solution feasible in the United States? (review article)	Technology-augmented contact-based surveillance is analyzed regarding their role in monitoring the COVID-19 outbreak.
Storeng, K. T. [57]	2021	The Smartphone Pandemic: How Big Tech and public health authorities' partner in the digital response to Covid-19 (review article)	The authors critically discuss new forms of public-private cooperation caused by the COVID-19 pandemic.
Sullivan, C. [58]	2021	Moving Faster than the COVID-19 Pandemic: The Rapid, Digital Transformation of a Public Health System (original research)	In this case study, the digitization of the public health system in Queensland, Australia, is analyzed regarding its benefits and lessons learnt during the COVID-19 pandemic.
Tejedor, S. [59]	2020	Tracking Websites' Digital Communication Strategies in Latin American Hospitals During the COVID-19 Pandemic (original research)	The highest-ranked hospitals in Latin America are analyzed regarding their websites during the COVID-19 pandemic.
Thomas, M. J. [60]	2021	Can technological advancements help to alleviate COVID-19 pandemic? a review (review article)	This review provides technological solutions which can be useful in combatting COVID-19.

Tiirinki, H [61]	2020	COVID-19 pandemic in Finland - Preliminary analysis on health system response and economic consequences (research article)	The authors analyze the effects of COVID-19 in Finland regarding health policy, the social- and health system and the economic system.
Whitelaw, S. [62]	2020	Applications of digital technology in COVID-19 pandemic planning and response (perspective article)	In this viewpoint, the authors describe a framework for applying digital technologies to combat COVID-19 and analyze how various countries have adopted these technologies.
Wood, A. [63]	2021	Linked electronic health records for research on a nationwide cohort of more than 54 million people in England: data resource (original research)	An electronic health record resource and its usefulness in fighting the COVID-19 pandemic is described.
Xu, X., Cai, Y. & Wu, S. [64]	2021	Assessment of Internet Hospitals in China During the COVID-19 Pandemic: National Cross-Sectional Data Analysis Study (original research)	The authors analyze China's internet hospitals and their health service capacity.
Yan, A. [65]	2020	How hospitals in mainland China responded to the outbreak of COVID-19 using information technology-enabled services: An analysis of hospital news webpages (original research)	The application and impact of information technology on Chinese hospitals are investigated.
Ye, Q. & Zhou, J. [66]	2020	Using Information Technology to Manage the COVID-19 Pandemic: Development of a Technical Framework Based on Practical Experience in China (original research)	The authors develop a framework to respond to COVID-19 from a health informatics perspective.
Yen, W. T. [67]	2020	Taiwan's COVID-19 Management: Developmental State, Digital Governance, and State-Society Synergy (review article)	In this review of policy practice, the authors analyze Taiwan's response strategy to COVID-19.
Zanin, M. [68]	2020	The public health response to the COVID-19 outbreak in mainland China: a narrative review (review article)	The authors analyze China's response strategy to COVID-19.
Zeng, K. & Bernardo, S. N. [69]	2020	The Use of Digital Tools to Mitigate the COVID-19 Pandemic: Comparative Retrospective Study of Six Countries (original research)	Precautions to combat COVID-19 in various countries are compared.
Zhao, Z. [70]	2021	Applications of Robotics, Artificial Intelligence, and Digital Technologies During COVID-19: A Review (review article)	News articles and scientific reports on technology adoption in combatting COVID-19 are analyzed.

Distribution of authors' residence

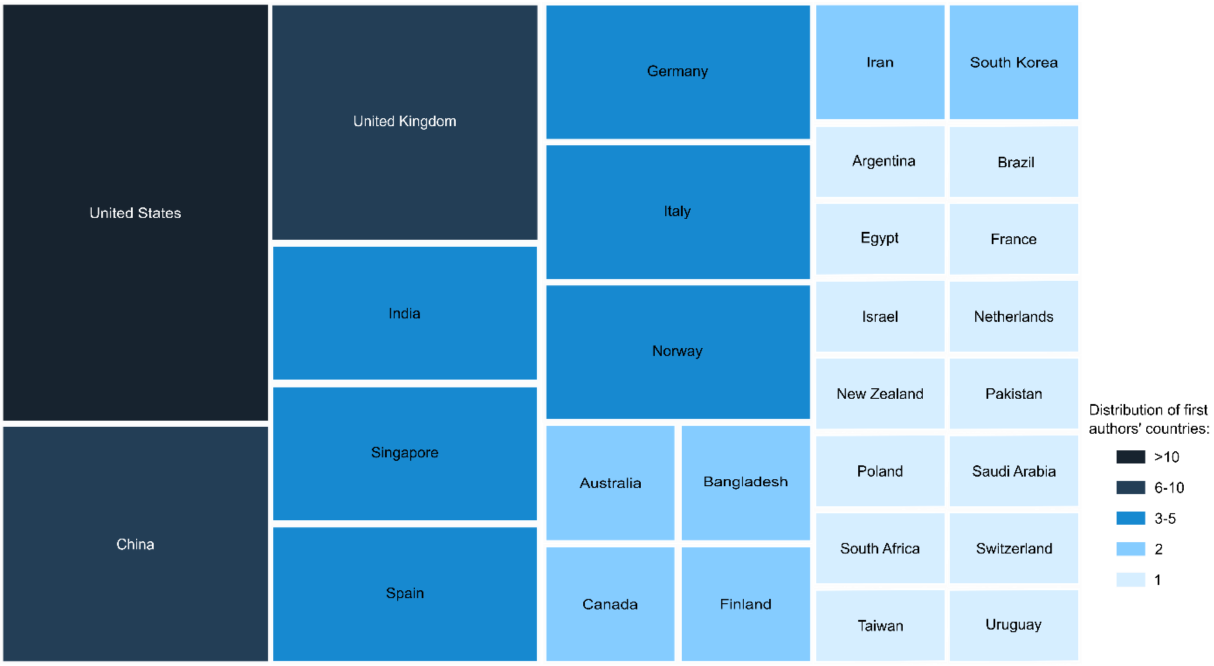

Supplementary Figure 4. The distribution of first authors' residence. This also includes multiple countries in the case of co-first authorship.

Distribution of countries evaluated in the included papers

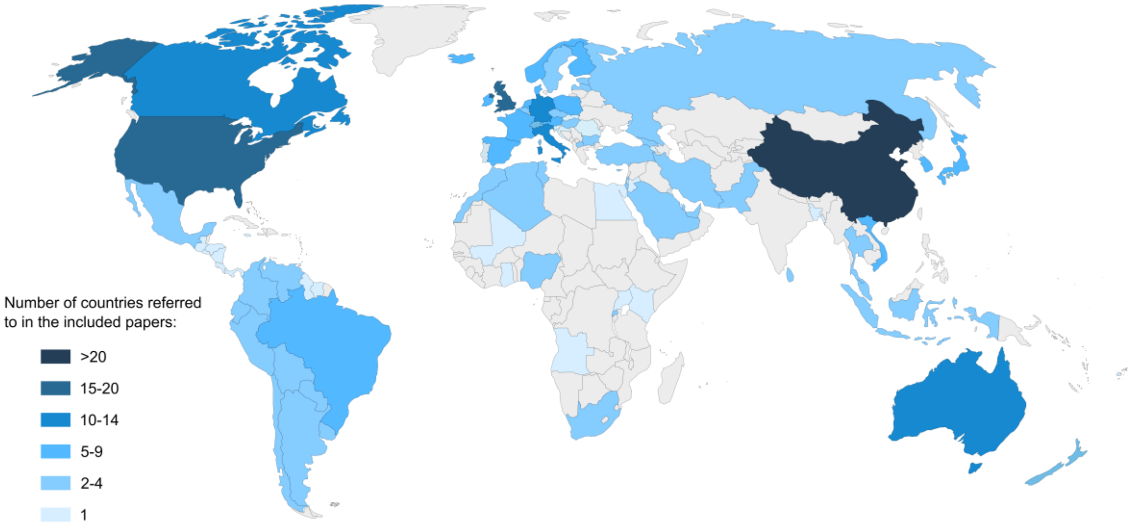

Supplementary Figure 5. Geographical map of screened reports. The distribution of countries evaluated in articles included in the scoping review. This map was adapted from https://de.wikipedia.org/wiki/Datei:BlankMap-World-v2.svg#file [71].

Data synthesis

Supplementary Table 5. Data synthesis. This matrix illustrates the occurrence of keywords and themes for each reference included in this scoping review.

		Healthcare system			Government response			Determinants of public health outcomes		
No.	First author	digital infrastructure	clinical managment	telehealth	contact tracing	non-pharmaceutical interventionss	evidence-based guidance	risk communication	sociocultural & political context	public attitudes
1	Abdel-Basset, M.	1					1			
2	Abueg, M. & Hinch, R.				1	1				
3	Aggarwal, L.						1			
4	Alexiadis, A.						1			
5	Allam, M.				1		1			
6	Almalki, A.				1					
7	Anthony, B.		1	1						
8	Anthony, B.	1	1	1					1	
9	Anttiroiki, A. V.					1			1	1
10	Asadzadeh, A.			1	1					
11	Basellini, U.				1	1				
12	Behar, J. A.	1		1						1
13	Boeing, P.				1					
14	Budd, J.				1			1	1	1
15	Cencetti, G. & Santin, G.				1	1	1			
16	Chatterjee, P.	1	1	1						
17	Chaudhary, Y.				1					
18	Elavarasan, R. M.						1			
19	Faraj, S.								1	1
20	Fariniuk, T. M. D.								1	
21	Ferretti, L. & Wymant, C.				1	1				
22	Golinelli, D.	1	1	1						
23	Gunasekeran, D. V.	1		1						
24	He, Z., Zhang, C. J. P., and Huang, J.				1	1	1			
25	Heo, K.					1				
26	Hernández-Orallo, E.				1		1			
27	Hernández-Orallo, E.				1		1			
28	Huang, Z.	1			1					
29	Islam, M. N., and Islam, A. K. M. N.					1				
30	Javaid, M.	1		1						

31	Javaid, M.	1	1	1						
32	Jian, S. W.				1					
33	Jung, S. Y.	1	1	1						
34	Kalhori, S. R. N.	1		1						
35	Khan, Z. H.	1	1							
36	Kogan, N.					1				
37	Król, K.	1						1		
38	Kucharski, A. J.				1	1	1			
39	Lai, S. H. S.				1					
40	Leung, K.						1			
41	Liaw, S. T.	1	1	1					1	
42	Lin, L.				1	1				
43	Mahdi, A.						1			
44	Martin, T.				1		1			
45	Meijer, A.		1		1	1		1	1	
46	Nachega, J. B.				1					
47	Nageshwaran, G.				1	1				
48	Nuzzo, A.				1	1				
49	O'Connor, H.				1	1				1
50	Reddick, C. G.								1	
51	Rodríguez, P.				1					
52	Salathé, M.				1					
53	Schlosser, F.					1				
54	Scott, B. K.	1	1	1						
55	Shamil, M. S.				1	1	1			
56	Skoll, D.				1	1				1
57	Storeng, K. T.				1			1		1
58	Sullivan, C.	1								
59	Tejedor, S.	1						1		
60	Thomas, M. J.							1		
61	Tiirinki, H.					1			1	
62	Whitelaw, S.	1	1	1	1	1			1	
63	Wood, A.	1								
64	Xu, X.	1		1						
65	Yan, A.	1	1	1				1		
66	Ye, Q.	1					1			
67	Yen, W. T.					1		1	1	1
68	Zanin, M.				1	1				
69	Zeng, K.				1	1				
70	Zhao, Z.	1	1	1						
	**Totals**	23	13	17	32	23	15	8	11	8

Note: Totals reported in the results section of the manuscript correspond to the number of papers to which at least one of the three keywords could be assigned per topic: i) healthcare system (n=26), ii) government responses (n=46), and iii) determinants facilitating or hindering public health outcomes (n=19).

Literature findings of the scoping review: Determinants facilitating or hindering public health outcomes
For the final theme of our scoping review, we combined the literature discussing influential factors that hindered or facilitated the early response to the pandemic and that do not directly relate to digital technologies used in clinical settings or by governments. Three keywords emerged under this theme: socioeconomic and political contexts, risk communication, and public attitudes. Supplementary Fig. 3 shows the occurrence and overlap of all nine keywords included in the scoping review. Large disparities in affordability and access to mobile communications widen the digital divide [14]. Low-income settings, ethnic minorities, less educated groups, and the digitally illiterate are disproportionally affected by digital inequalities [50]. Reddick et al. illustrate this with a case study of broadband access in San Antonio, USA, where more than half the population is Hispanic. Residents of poorer neighborhoods had significantly lower rates of broadband use, defined here as an essential factor in a person's quality of life. This study highlights the significant digital divide within geographic structures [50]. Anthony et al. identified barriers to adopting digital health solutions at the infrastructural and institutional level, similar to the DAI pillars. They ultimately created determinants meaningful in adopting telehealth [8]. In addition, great exposure to information, inappropriate risk communication, or even misinformation have confused and influenced individual health behaviors. Misinformation and changing guidance have led people to reject public health measures such as wearing masks and physical distancing [14,60]. Finally, digital technologies raise legal, ethical, and privacy concerns [8,57].

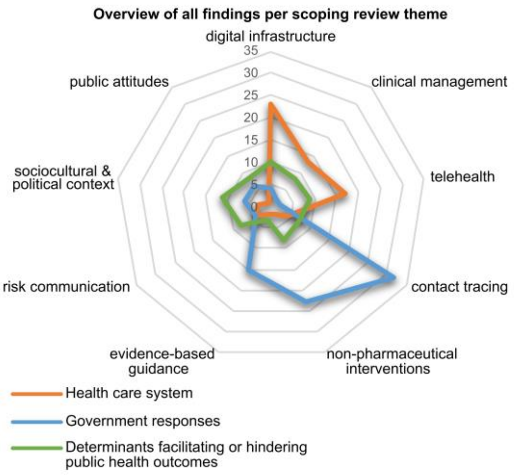

Supplementary Figure 6. Occurrence of keywords and themes within the scoping review. The radar chart illustrates the frequency and overlap of studies assigned to the following three themes of the scoping review: i) health care system, ii) government responses, and iii) determinants facilitating or hindering public health outcomes. The results of the first two themes are extensively described in the main manuscript. The third theme (green) includes keywords from all three themes. The axis of the radar chart represents the total number of included keywords within the themes (see Supplementary Table 5). The scale for the radial aces of this chart is the total number of reports per keyword.

Global level of digital adoption by income

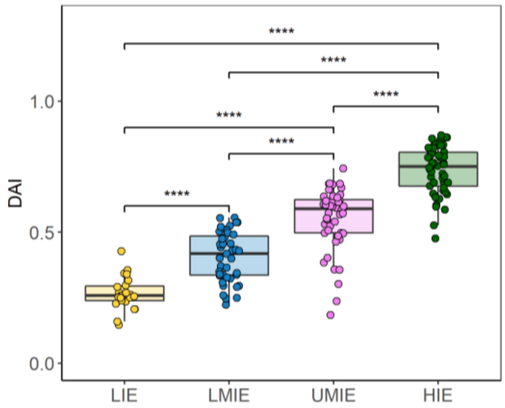

Supplementary Figure 7. Distribution of digital adoption by income group. Box and Whisker plot of the digital adoption index (DAI) by income level: low, lower-middle, upper-middle, and high income. One-way ANOVA of income groups followed by a Games-Howell post-hoc test for pairwise comparisons. DAI showed statistically significant differences between all groups ($p<0{\cdot}001$). (Note: LIE = low-income economy; LMIE = lower-middle-income economy; UMIE = upper-middle-income economy; HIE = high-income economy).

Supplementary Table 6. Post-hoc comparison of means between income groups.

group 1	group 2	t	p-value (adj.)
HIE	LIE	-36.532	0.0001*
HIE	LMIE	-24.341	0.0001*
HIE	UMIE	-11.965	0.0001*
LIE	LMIE	10.246	0.0001*
LIE	UMIE	18.187	0.0001*
LMIE	UMIE	8.889	0.0001*

* $p<0.0001$

Supplementary References

This reference list also includes references to all studies included in the scoping review.

1 Abdel-Basset, M., Chang, V. & Nabeeh, N. A. An intelligent framework using disruptive technologies for COVID-19 analysis. *Technol. Forecast. Soc. Change* **163**, 120431 (2021).

2 Abueg, M. *et al.* Modeling the effect of exposure notification and non-pharmaceutical interventions on COVID-19 transmission in Washington state. *NPJ Digit. Med.* **4**, 49 (2021).

3 Aggarwal, L., Goswami, P. & Sachdeva, S. Multi-criterion Intelligent Decision Support system for COVID-19. *Appl. Soft. Comput.* **101**, 107056 (2021).

4 Alexiadis, A. *et al.* Simulation of pandemics in real cities: enhanced and accurate digital laboratories. *Proc. Math. Phys. Eng. Sci.* **477**, 20200653 (2021).

5 Allam, M. *et al.* COVID-19 Diagnostics, Tools, and Prevention. *Diagnostics (Basel)* **10**, 409 (2020).

6 Almalki, M. & Giannicchi, A. Health Apps for Combating COVID-19: Descriptive Review and Taxonomy. *JMIR Mhealth Uhealth* **9**, e24322 (2021).

7 Anthony, B. Integrating telemedicine to support digital health care for the management of COVID-19 pandemic. *Int. J. Healthc. Manag.* **14**, 280-289 (2021).

8 Anthony Jnr, B. Implications of telehealth and digital care solutions during COVID-19 pandemic: a qualitative literature review. *Inform. Health. Soc. Care* **46**, 68-83 (2021).

9 Anttiroiko, A. V. Successful Government Responses to the Pandemic: Contextualizing National and Urban Responses to the COVID-19 Outbreak in East and West. *International Journal of E-Planning Research* **10**, 1-17 (2021).

10 Asadzadeh, A. & Kalankesh, L. R. A scope of mobile health solutions in COVID-19 pandemics. *Inform. Med. Unlocked* **23**, 100558 (2021).

11 Basellini, U. *et al.* Linking excess mortality to mobility data during the first wave of COVID-19 in England and Wales. *SSM Popul. Health* **14**, 100799 (2021).

12 Behar, J. A. *et al.* Remote health diagnosis and monitoring in the time of COVID-19. *Physiol. Meas.* **41** (2020).

13 Boeing, P. & Wang, Y. H. Decoding China's COVID-19 'virus exceptionalism': Community-based digital contact tracing in Wuhan. *R & D Management* **51**, 339-351 (2021).

14 Budd, J. *et al.* Digital technologies in the public-health response to COVID-19. *Nat. Med.* **26**, 1183-1192 (2020).

15 Cencetti, G. *et al.* Digital proximity tracing on empirical contact networks for pandemic control. *Nat. Commun.* **12**, 1655 (2021).

16 Chatterjee, P., Tesis, A., Cymberknop, L. J. & Armentano, R. L. Internet of Things and Artificial Intelligence in Healthcare During COVID-19 Pandemic-A South American Perspective. *Front. Public Health* **8**, 600213 (2020).

17 Chaudhary, Y., Sandhu, N., Singh, A., Aggarwal, P. & Naithani, M. Digital Warfare Against COVID-19: Global Use of Contact-Tracing Apps. *Asia Pac. J. Public Health* **33**, 945-948 (2021).

18 Elavarasan, R. M., Pugazhendhi, R., Shafiullah, G. M., Irfan, M. & Anvari-Moghaddam, A. A hover view over effectual approaches on pandemic management for sustainable cities – The endowment of prospective technologies with revitalization strategies. *Sustain. Cities Soc.* **68**, 102789 (2021).

19 Faraj, S., Renno, W. & Bhardwaj, A. Unto the breach: What the COVID-19 pandemic exposes about digitalization. *Information and Organization* **31**, 100337 (2021).

20 Fariniuk, T. M. D. Smart cities and the pandemic: digital technologies on the urban management of Brazilian cities. *Revista De Administracao Publica* **54**, 860-873 (2020).

21 Ferretti, L. *et al.* Quantifying SARS-CoV-2 transmission suggests epidemic control with digital contact tracing. *Science* **368** (2020).

22 Golinelli, D. *et al.* Adoption of Digital Technologies in Health Care During the COVID-19 Pandemic: Systematic Review of Early Scientific Literature. *J. Med. Internet Res.* **22**, e22280 (2020).

23 Gunasekeran, D. V., Tseng, R., Tham, Y. C. & Wong, T. Y. Applications of digital health for public health responses to COVID-19: a systematic scoping review of artificial intelligence, telehealth and related technologies. *NPJ Digit. Med.* **4**, 40 (2021).

24 He, Z. *et al.* A New Era of Epidemiology: Digital Epidemiology for Investigating the COVID-19 Outbreak in China. *J. Med. Internet Res.* **22**, e21685 (2020).

25 Heo, K., Lee, D., Seo, Y. & Choi, H. Searching for Digital Technologies in Containment and Mitigation Strategies: Experience from South Korea COVID-19. *Ann. Glob. Health* **86**, 109 (2020).

26 Hernandez-Orallo, E., Calafate, C. T., Cano, J. C. & Manzoni, P. Evaluating the Effectiveness of COVID-19 Bluetooth-Based Smartphone Contact Tracing Applications. *Applied Sciences-Basel* **10** (2020).
27 Hernandez-Orallo, E., Manzoni, P., Calafate, C. T. & Cano, J. C. Evaluating How Smartphone Contact Tracing Technology Can Reduce the Spread of Infectious Diseases: The Case of COVID-19. *Ieee Access* **8**, 99083-99097 (2020).
28 Huang, Z. *et al.* Performance of Digital Contact Tracing Tools for COVID-19 Response in Singapore: Cross-Sectional Study. *JMIR Mhealth Uhealth* **8**, e23148 (2020).
29 Islam, M. N. & Islam, A. A Systematic Review of the Digital Interventions for Fighting COVID-19: The Bangladesh Perspective. *Ieee Access* **8**, 114078-114087 (2020).
30 Javaid, M. *et al.* Industry 4.0 technologies and their applications in fighting COVID-19 pandemic. *Diabetes Metab. Syndr.* **14**, 419-422 (2020).
31 Javaid, M. *et al.* Industry 5.0: Potential Applications in COVID-19. *J. Ind. Inf. Integr.* **5**, 507-530 (2020).
32 Jian, S. W., Cheng, H. Y., Huang, X. T. & Liu, D. P. Contact tracing with digital assistance in Taiwan's COVID-19 outbreak response. *Int. J. Infect. Dis.* **101**, 348-352 (2020).
33 Jung, S. Y., Lee, H. Y., Hwang, H., Lee, K. & Baek, R. M. How IT preparedness helped to create a digital field hospital to care for COVID-19 patients in S. Korea. *NPJ Digit. Med.* **3**, 157 (2020).
34 Kalhori, S. R. N. *et al.* Digital Health Solutions to Control the COVID-19 Pandemic in Countries With High Disease Prevalence: Literature Review. *J. Med. Internet Res.* **23**, e19473 (2021).
35 Khan, Z. H., Siddique, A. & Lee, C. W. Robotics Utilization for Healthcare Digitization in Global COVID-19 Management. *Int. J. Environ. Res. Public Health* **17** (2020).
36 Kogan, N. E. *et al.* An early warning approach to monitor COVID-19 activity with multiple digital traces in near real time. *Sci. Adv.* **7** (2021).
37 Król, K. & Zdonek, D. The Quality of Infectious Disease Hospital Websites in Poland in Light of the COVID-19 Pandemic. *Int. J. Environ. Res. Public Health* **18** (2021).
38 Kucharski, A. J. *et al.* Effectiveness of isolation, testing, contact tracing, and physical distancing on reducing transmission of SARS-CoV-2 in different settings: a mathematical modelling study. *Lancet Infect. Dis.* **20**, 1151-1160 (2020).
39 Lai, S. H. S., Tang, C. Q. Y., Kurup, A. & Thevendran, G. The experience of contact tracing in Singapore in the control of COVID-19: highlighting the use of digital technology. *Int. Orthop.* **45**, 65-69 (2021).
40 Leung, K., Wu, J. T. & Leung, G. M. Real-time tracking and prediction of COVID-19 infection using digital proxies of population mobility and mixing. *Nat. Commun.* **12**, 1501 (2021).
41 Liaw, S. T. *et al.* Primary Care Informatics Response to Covid-19 Pandemic: Adaptation, Progress, and Lessons from Four Countries with High ICT Development. *Yearb. Med. Inform.* **30**, 44-55 (2021).
42 Lin, L. & Hou, Z. Y. Combat COVID-19 with artificial intelligence and big data. *J. Travel Med.* **27** (2020).
43 Mahdi, A. *et al.* OxCOVID19 Database, a multimodal data repository for better understanding the global impact of COVID-19. *Sci. Rep.* **11**, 9237 (2021).
44 Martin, T. *et al.* Demystifying COVID-19 Digital Contact Tracing: A Survey on Frameworks and Mobile Apps. *Wireless Communications & Mobile Computing* **2020** (2020).
45 Meijer, A. & Webster, C. W. R. The COVID-19-crisis and the information polity: An overview of responses and discussions in twenty-one countries from six continents. *Information Polity* **25**, 243-274 (2020).
46 Nachega, J. B. *et al.* Contact Tracing and the COVID-19 Response in Africa: Best Practices, Key Challenges, and Lessons Learned from Nigeria, Rwanda, South Africa, and Uganda. *Am. J. Trop. Med. Hyg.* **104**, 1179-1187 (2021).
47 Nageshwaran, G., Harris, R. C. & Guerche-Seblain, C. E. Review of the role of big data and digital technologies in controlling COVID-19 in Asia: Public health interest vs. privacy. *Digit. Health* **7**, 20552076211002953 (2021).
48 Nuzzo, A. *et al.* Universal Shelter-in-Place Versus Advanced Automated Contact Tracing and Targeted Isolation: A Case for 21st-Century Technologies for SARS-CoV-2 and Future Pandemics. *Mayo Clin. Proc.* **95**, 1898-1905 (2020).
49 O'Connor, H., Hopkins, W. J. & Johnston, D. For the greater good? Data and disasters in a post-COVID world. *Journal of the Royal Society of New Zealand* **51**, 214-231 (2021).
50 Reddick, C. G., Enriquez, R., Harris, R. J. & Sharma, B. Determinants of broadband access and affordability: An analysis of a community survey on the digital divide. *Cities* **106**, 102904 (2020).
51 Rodríguez, P. *et al.* A population-based controlled experiment assessing the epidemiological impact of digital contact tracing. *Nat. Commun.* **12**, 587 (2021).
52 Salathé, M. *et al.* Early evidence of effectiveness of digital contact tracing for SARS-CoV-2 in Switzerland. *Swiss. Med. Wkly.* **150**, w20457 (2020).

53 Schlosser, F. *et al.* COVID-19 lockdown induces disease-mitigating structural changes in mobility networks. *PNAS* **117**, 32883-32890 (2020).
54 Scott, B. K. *et al.* Advanced Digital Health Technologies for COVID-19 and Future Emergencies. *Telemed. J. E. Health* **26**, 1226-1233 (2020).
55 Shamil, M. S., Farheen, F., Ibtehaz, N., Khan, I. M. & Rahman, M. S. An Agent-Based Modeling of COVID-19: Validation, Analysis, and Recommendations. *Cognit. Comput.*, 1-12 (2021).
56 Skoll, D., Miller, J. C. & Saxon, L. A. COVID-19 testing and infection surveillance: Is a combined digital contact-tracing and mass-testing solution feasible in the United States? *Cardiovasc. Digit. Health J.* **1**, 149-159 (2020).
57 Storeng, K. T. & de Bengy Puyvallée, A. The Smartphone Pandemic: How Big Tech and public health authorities partner in the digital response to Covid-19. *Glob. Public Health* **16**, 1482-1498 (2021).
58 Sullivan, C. *et al.* Moving Faster than the COVID-19 Pandemic: The Rapid, Digital Transformation of a Public Health System. *Appl. Clin. Inform.* **12**, 229-236 (2021).
59 Tejedor, S., Pérez-Escoda, A., Ventín, A., Tusa, F. & Martínez, F. Tracking Websites' Digital Communication Strategies in Latin American Hospitals During the COVID-19 Pandemic. *Int. J. Environ. Res. Public Health* **17** (2020).
60 Thomas, M. J. *et al.* Can technological advancements help to alleviate COVID-19 pandemic? a review. *J. Biomed. Inform.* **117**, 103787 (2021).
61 Tiirinki, H. *et al.* COVID-19 pandemic in Finland - Preliminary analysis on health system response and economic consequences. *Health Policy Technol.* **9**, 649-662 (2020).
62 Whitelaw, S., Mamas, M. A., Topol, E. & Van Spall, H. G. C. Applications of digital technology in COVID-19 pandemic planning and response. *Lancet Digit. Health* **2**, e435-e440 (2020).
63 Wood, A. *et al.* Linked electronic health records for research on a nationwide cohort of more than 54 million people in England: data resource. *Bmj* **373**, n826 (2021).
64 Xu, X. *et al.* Assessment of Internet Hospitals in China During the COVID-19 Pandemic: National Cross-Sectional Data Analysis Study. *J. Med. Internet Res.* **23**, e21825 (2021).
65 Yan, A., Zou, Y. & Mirchandani, D. A. How hospitals in mainland China responded to the outbreak of COVID-19 using information technology-enabled services: An analysis of hospital news webpages. *J. Am. Med. Inform. Assoc.* **27**, 991-999 (2020).
66 Ye, Q., Zhou, J. & Wu, H. Using Information Technology to Manage the COVID-19 Pandemic: Development of a Technical Framework Based on Practical Experience in China. *JMIR Med. Inform.* **8**, e19515 (2020).
67 Yen, W. T. Taiwan's COVID-19 Management: Developmental State, Digital Governance, and State-Society Synergy. *Asian Politics & Policy* **12**, 455-468 (2020).
68 Zanin, M. *et al.* The public health response to the COVID-19 outbreak in mainland China: a narrative review. *J. Thorac. Dis.* **12**, 4434-4449 (2020).
69 Zeng, K., Bernardo, S. N. & Havins, W. E. The Use of Digital Tools to Mitigate the COVID-19 Pandemic: Comparative Retrospective Study of Six Countries. *JMIR Public Health Surveill.* **6**, e24598 (2020).
70 Zhao, Z. *et al.* Applications of Robotics, Artificial Intelligence, and Digital Technologies During COVID-19: A Review. *Disaster Med. Public Health Prep.*, 1-11 (2021).
71 *Wikipedia*. https://de.wikipedia.org/wiki/Datei:BlankMap-World-v2.svg#file (2012).
